# Asiatic Cholera

**Published:** 1885-08

**Authors:** 


### ASIATIC CHOLERA.

The invasion of France and Italy by Asiatic cholera in 1884, and its greater prevalence in Spain at the present moment, give rise to reasonable apprehension that it will shortly re-visit the United States. Indeed, in view of its past history, it may be looked for in some one or more of our seaport towns at any time.

Cholera appeared first in the United States in 1802, and since then in 1848, 1854, 1867 and in 1873. In no instance has it been shown to be of local origin, but in every case has been clearly traced to one or more ports in Europe from which it has been brought to America. Every epidemic here has been preceded by one more or less extensive in Europe, and every outbreak of it in Europe has been traced back through a continuous chain of human beings to its original seat in Hindostan.

From this common origin all the epidemics of cholera which have afflicted the nations have spread by slow and uncertain steps in all directions along the lines of human intercourse. No conditions of temperature, of geological formation, of atmosphere or of elevation will stay its progress. It prevails in summer or winter, in wet or dry seasons. It follows the navigator on his ocean voyages and the traveler over sterile plains or mountain passes.

Our first epidemic began in India in 1827. Following caravans it crossed the Caucassian heights, spread over Persia, around the Caspian sea, and reached Russia in 1829. It was conveyed to Sunderland in England in 1831. It quickly spread over Europe and came to America by different routes—reaching Quebec from Dublin and New York from London about the same time in 1832.

Some of the subsequent epidemics have reached Europe

(always from the original nidus) through different channels, and have invaded the United States at different points. New Orleans had the first cases in 1848, and in 1867 the latter brought directly from some Mediterranean port, or indirectly from Savannah.

The epidemic, which at this time excites interest and apprehension, reached Egypt in 1883, and during the summer of that year spread generally over the country. Great care was taken to prevent its passage into Europe, but despite the precautions employed a case occurred in Toulon, in France, in June, 1884. It soon became epidemic there, also in Marseilles and in other places. Thence it was conveyed to Italy, and during the year cost those two countries probably fifty thousand lives and hundreds of millions of money.

The quarantine on the Spanish frontier was exceedingly severe, but by some means it was evaded and a few cases introduced into Spain, where, in this year, 1885, it still prevails with great fatality.

This brief synopsis of the history of former epidemics, as well as a more careful study of the diseases, justify a few general conclusions:

1. The specific poison which produces cholera originates alone in India, and is transmitted through the persons of infected individuals, or by infected fabrics, to all quarters of the world. It has never appeared upon this continent, but after the arrival of infected persons with their property from infected districts.

2. The spread of cholera when once introduced into a country is unequal, partial and erratic. It never travels faster than man; it attaches itself to his person and follows his footsteps; currents of wind neither hasten nor retard it.

3. The inhabitants of certain towns or districts are especially liable to be visited by it, while others escape its influence. Some places it attacks with extreme severity, while others suffer very slightly. This difference is not satisfactorily explained by differences in their general salubrity, geological formation or sanitary condition of the inhabitants.

4. The intensity of cholera varies during its continuance in a country or town, and has generally well marked periods of increase, culmination and decline.

5. After having been for an uncertain time epidemic in a locality it entirely disappears. In this country, in particular localities, it has lingered with varying intensity for one or two years before its final cessation.

No known physical laws give an explanation of this important fact. Why the malignant vigor of the poison should terminate is no better known than the reason why its origin is restricted to Hindostan. The ultimate fact is consolatory in view of the probable advent of a great evil.

6. One attack of cholera gives to the individual no immunity from the disease in future.

In epidemics of cholera in countries or cities, only a small portion of the inhabitants suffer from it, but its victims are not confined to any age, sex or conditions of society. The proportion of deaths to the whole number of cases varies greatly in different localities and in different epidemics. As a general rule its fatality is greatest at the outbreak of the attack. The severity of an epidemic is by no means proportioned to its territorial extent, and the slower its progress the less its fatality. For this reason it may reasonably be hoped that, should the disease reach America this year or the next, it will be less destructive than in countries where it now prevails.

The study of the epidemic of 1832 gave to the profession full information in relation to the natural history of the disease, as well as to its morbid anatomy and pathological history, but left its etiology, the means of its prevention and cure, shrouded in the darkness of theories, conjecture and speculation.

The more recent epidemics have added but little that is satisfactory to the sum of our knowledge.

Intelligent inquiry is still directed to the subject, and the world hopefully awaits the result.

It is too early to speak dogmatically of these recent investigations, as they are still in progress; greatest interest clusters around the discussion of the old parasite theory of the causation of the disease, and the microscopic observations of the parasites or germs supposed to produce it. Our space does not permit even a synopsis of this discussion. By many it is hoped that we are very near to the discovery of the precise organism causation of cholera, and also of the means of destroying it and preventing its devastation. As yet, that point has not been attained. Bacilli of various shapes found in the dejecta of cholera patients have been described, depicted and named, but in the very theatre of the observations and experiments, the old average of fifty per cent. of deaths of the number attacked still nourishes doubt and apprehension.

Encouraged by the brilliant success of M. Pasteur, in France, in preventing a destructive disease of sheep, Koch and others are attempting to prevent the cholera by the same method, viz.: by inducing a modified form of the disease, by inoculating the wells with an attenuated virus prepared from cholera dejecta. What will be the result of these experiments cannot be foretold; only newspaper accounts of them have reached us; these, to say the least of them, are not encouraging. It is stated that many of the persons inoculated become ill, have symptoms resembling cholera for a few days, and recover; others have produced bad ulcers at the point of inoculation, inducing a suspicion of septicemia.

As to its protective power, in view of the fact that genuine cholera does not prevent a second attack of the same disease, it is not claimed that the illness produced by the Koch inoculation will protect the inoculated longer than its continuance; this, by some observers, is stated to be six days, by others two weeks. As it is impossible to inoculate an entire community every two weeks, the prospect is not encouraging.

---

### ITEMS.

DR. J. C. LEHARDY, of Savannah, who has been quite sick for several weeks, has entirely recovered.

DR. M. ALLEN STARR has been made a Professor of Diseases of the Mind and Nervous System in the New York Polyclinic.

DR. W. P. HARDEN, of Harmony Grove, a member of the Medical Association of Georgia since 1853, died a few weeks since.

DR. W. G. BULLOCK, of Savannah, ex-President of the Medical Association of Georgia, died at his home in Savannah a few weeks since.

DR. ROBERT BATTEY, of Rome, was made President of the Section on Gynecology by General Committee on the Organization of the International Medical Congress, held at Chicago June 25th.

DR. HENRY WILE, late Clinical Assistant in Dermatology in the University of Pennsylvania, and Private Assistant to Professor L. A. Duhring, of Philadelphia, has located in Atlanta and will devote himself entirely to the practice of diseases of the skin.

MISSED HIM.—One of the most prominent physicians of Indianapolis, on his return from a summer vacation of three weeks, was met by the leading undertaker, who said: "Ah, doctor, so glad to see you safe back; we have missed you so much."—*Ex.*

THE city physicians of Savannah are paid a salary of $50.00 per month; those of Atlanta are paid from $26.00 to $35.00 per month.

DR. MARK H. O'DANIEL, of Milledgeville, says: "I am delighted with the publication of the Transactions of the Association in THE JOURNAL."

WELL DONE.—The city of Chicago has appropriated $100,000 to the Health Department. Of this amount $12,000 will be expended on the inspectors by increasing the force. The sum of $40,000 will be spent in cleaning of alleys, and the remainder will be held for an emergency fund.

THE FAMILY DOCTOR.—Little Boy: "Please I want the doctor to come and see mother." Servant: "Doctor's out. Where do you come from?" Little Boy: "What! Don't you know me? Why, we deal with you. We had a baby from here last week!"

DR. O. G. DARLING, of Brooklyn, N. Y., in the *Therapeutic Gazette*, claims that mur. of ammonia in half drachm doses, every half hour, if necessary, until three or four doses have been taken, is a specific for facial neuralgia. He is in the habit of continuing the remedy in smaller doses, say ten grains, three or four times a day for a day or two after the neuralgia subsides. It is also valuable for toothache.

ASYLUM TRAGEDIES.—News comes from Marseilles of a sad occurrence in the Saint Pierre Lunatic Asylum. A keeper put two patients each in a separate bath, left them and went to breakfast. One of the lunatics got out of his bath, during the absence of the keeper, and turned on the tap of hot water into the bath of the other. When the keeper returned he found that the unhappy creature had been scalded to death. In the Asylum at Sebon, near Dinau, a lunatic suddenly assaulted one of the attendants; another attendant who started to the rescue was attacked by a second lunatic. Both the attendants were knocked down and one mortally wounded. The two homicides then started for the central building with the intention of killing the superintendent. They were, however, captured and secured.—*Medical Record.*

NIGHT-SWEATS.—Prof. DaCosta says that in the treatment of the night-sweats of phthisis ergot is preferable to atropine, being more permanent in its effects, and has the additional merit of not producing the dryness of the throat which so frequently follows the administration of atropine. He would give gr. ij. of ergotin ter die, the last dose given at bed time.—*College and Clinical Record.*

THE attention of our readers is especially directed to the advertisements contained in the THE ATLANTA MEDICAL AND SURGICAL JOURNAL. By reading them they are informed of many matters of interest and importance to the profession. In corresponding with advertisers, please mention THE JOURNAL.

WE desire to call attention to the advertisement elsewhere of Haywood White Sulphur Springs, near Waynesville, North Carolina. There is no better climate in America for consumptives than the climate of western North Carolina. This is verified by the new climatic map just issued by Dr. Denison, of Colorado.

SPECIFIC BACILLI IN SYPHILITIC TISSUES.—The characteristic bacilli of syphilis seem to have been at last discovered. Dr. S. Sustgarten (*Centrlbl. f. d. Med. Wissensch.*, 9, 1885) has demonstrated in two cases of initial syphilitic sclerosis, and in one case of a gumma, the presence of bacilli, which by their reaction to a peculiar method of coloring, and by other properties, were proven to be a separate species, thus far not described. Koch and Weigert have confirmed the discovery. The bacilli great[illegible] resemble in size and shape those of tubercle, but they are alw[illegible]

met with either singly or in small groups, included in slightly swollen lymphoid cells. Under high power they showed the same light spots which Koch believes to be spore-formations. Cocci were not present.

THE TREATMENT OF HEMORRHOIDS BY INJECTION.—In an instructive clinical paper in the July number of the *American Journal of the Medical Sciences*, Dr. Charles B. Kelsey, of New York, urges the treatment of hemorrhoids by injection of carbolic acid. After an ample experience this has become his routine practice, and in all his cases he has never known a patient to abandon the treatment after it was begun, and he has never failed to effect a perfectly satisfactory cure by it, and he has never had an accident of serious nature with it.

He uses three solutions, one of 15 per cent., one of 33 per cent., one of 50 per cent., and sometimes he uses the pure acid. In a severe case he begins with the stronger ones, in a mild case with the weaker.

THE *Medical and Surgical Reporter* says Koch's "comma bacillus" appears to be having a hard time of it among the experts. First, Dr. Klein showed his contempt of it by swallowing it, and now Dr. Lancaster has the unkindness to say, first, it is not comma-shaped; second, it is not a bacillus; third, it does not always occur in the intestines of cholera patients; and fourth, there is no good evidence that inoculation with it produces cholera. In fact, the poor thing appears to be about annihilated by its critics. Cholera, though, will remain undisturbed by it, and relentlessly claim its thousands of victims as heretofore.

REMARKABLE LONGEVITY.—The time is not far distant when the possibility of human life being prolonged in the present day to the age of one hundred years was denied by some. Satisfactory proofs to the contrary have, however, been frequently furnished; and the return issued last month by the Registrar-General of Scotland for the first quarter of the present year affords further instances, for in it there are recorded the deaths of no fewer than three centenarians. The oldest of these was a man, who died in Nairn at the patriarchal age of 104 years, while the other two were women, aged respectively 102 and 101 years, the elder of the two dying at Kirkintilloch, the younger in the Gairloch district of Ross and Cromarty. Side by side with these well authenticated cases of long life, we find reported from the Glengairn district of Aberdeenshire the deaths of three females, whose united age amounted to 264 years, the oldest of them being 98.—*Brit. Med. Jour.*

PEPTONIZED COD LIVER OIL AND MILK.—Dr. Wm. McGeachy, of Iona, reports having used with good results Peptonized Cod Liver Oil and Milk in the following cases, viz.:

1. Strumous enlargement of the glands of the neck.
2. Post-nasal catarrh with chronic tonsilitis.
3. The sequelæ of acute diseases.

He adds that as a remedy for the latter stages, and subsequent results of whooping-cough in children, it excels all remedies previously tried, and that in the second class of cases mentioned, it worked particularly well. He also found that the preparation was so highly peptonized as to act as a good digestive agent.—*Canadian Practitioner.*

THE following members of the Medical Association of Georgia occupy places on sections in the International Medical Congress as organized in Chicago: Section of Gynecology, Dr. Robert Battey of Rome, President; Section of Medical Education and Registration, Dr. J. W. Bailey of Gainesville; Section of Physiology, Dr. H. F. Campbell of Augusta; Section of Medicine, Dr. J. P. Logan of Atlanta; Section of Surgery, Dr. W. F. Westmoreland of Atlanta; Section of Collective Investigation, Nomenclature, Vital Statistics and Climatology, Dr. T. S. Hopkins of Thomasville; Section of Military and Naval Surgery and Medicine, Dr. J. McF. Gaston of Atlanta; Section of Diseases of Children, Dr. W. H. Doughty of Augusta, and Dr. W. F. Holt cf Macon, Ga.; Section of Ophthalmology, Dr. A. W. Calhoun of Atlanta.

CHLORAL HYDRATE IN EPILEPSY.—This valuable remedy is well known to the medical profession, but it may not be so generally known as it ought to be that it is sometimes of invaluable service in arresting epileptic fits, especially that form known as the *status epilepticus.* We have recently had some experience of its use in a case where all other remedies had failed, including inhalation of ether, chloroform and amyl nitrite. A twenty grain dose immediately put a stop to the frequently-recurring attacks, and the patient made a good recovery from the seizure.—*The Canada Lancet.*

THE Columbus *Medical Journal* for June, in commenting on the action of the American Medical Association toward the original Committee on the International Congress, says: The Committee had made no provision for the eloquent champion of the pap-chewing mothers of Texas; they had ignored the oleates; they had not tested all their appointees by the *shibboleth* of the Code; they had not given the "rural deestricts" their *numerical* ratio of the offices; whole States and Territories were entirely ignored; even Ohio had but fourteen, and of these her metropolis had all but one, while New York, Philadelphia and Boston were liberally remembered. And so there was a kick. And so it was decided to increase the size of the Committee, and otherwise so arrange matters that the ranches of Texas, the prairies of Kansas, the mines of Colorado, and the forests and wheat fields of the great Northwest should all be "represented" among the officers of this Congress.

Verily, we trust the original Committee, and their rustic accessories, may duly exercise the former patience, the latter humility, and both moderation, to the end that the Congress may be a success; for unless they do this we greatly apprehend the "kick" will overturn "the fat into the fire."

THE INTERNATIONAL MEDICAL CONGRESS AND THE MEDICAL PROFESSION OF BOSTON.—At a meeting of a number of members of the medical profession of Boston, which was held on Thursday, July 2d, the following preamble and resolution were adopted:

WHEREAS, We had been led to believe that the authority to organize and control the Ninth International Medical Congress had been permanently delegated by the American Medical Association to its original committee, thus providing against any radical changes in its published programme; and

WHEREAS, The American Medical Association has revised the action and annulled appointments of that committee in a way which we regard as detrimental to the interests of the medical profession of America and fatal to the success of the Congress; therefore, be it

*Resolved*, That we, the undersigned, members of the medical profession in Boston and vicinity, concerned in the organization of the Ninth International Medical Congress, decline to hold any office in said Congress as now organized:

| | |
|---|---|
| ROBERT AMORY, | GEORGE M. GARLAND, |
| CLARENCE J. BLAKE, | J. ORNE GREEN, |
| HENRY P. BOWDITCH. | FREDERICK I. KNIGHT, |
| JAMES R. CHADWICK, | FRANCIS MINOT, |
| HASKET DERBY, | OLIVER F. WADSWORTH, |
| THOMAS DWIGHT, | J. COLLINS WARREN, |
| ROBERT T. EDES, | SAMUEL G. WEBBER, |
| REGINALD H. FITZ, | E. WIGGLESWORTH, |
| CHARLES F. FOLSOM, | HENRY W. WILLIAMS, |

H. P. WOLCOTT.

A CASE OF CHOLECYSTOTOMY.—The rather recent revival of operative procedures for the relief of obstruction of the biliary ducts has awakened a new and lively interest in all such cases, especially as the entire number of reported operations falls within a total of fifty. To this list Dr. Charles T. Parkes, of Chicago, in the July number of the *American Journal of the Medical Sciences*, adds a very instructive case:

Of the cases submitted to operations, by far the larger portion has been of cholecystotomy, by means of which a distended gall-bladder has been positively relieved, and in most of them the cause of obstruction, retained gall-stones, removed. In a few no gall-stones were found, while in others the cause of the obstruction could not be remedied.

In Dr. Parkes' case the gall-stones were not lodged in the gall-bladder, and it is highly probable that they were retained in some of the dilated hepatic ducts, and were washed into the cyst by the flow of biliary fluid which came on immediately after the formation of the fistula. He is inclined to believe that they had but little to do with preventing the flow of bile into the intestine; at least the patulency of the passages was not restored by their removal. The remarkable and profuse flow of muco-biliary fluid, coming through the sinus established, argues very strongly in favor of the operation which contemplates the formation of a fistula in the gall-bladder in distension thereof by confined secretions. Certainly no tube of the dimensions of the ductus communis choledochus could have given a free exit to the secretions; at least the accompanying back pressure on the walls of the cyst would have greatly endangered, if not certainly destroyed, the adhesion in any recent wound thereof, even when protected by the continued suture. Most of the cases of reported cholecystotomy tell of the presence of a freer flow through the fistula, so that it is a condition to be expected, and makes immediate closure of the bladder wound without drainage a dangerous proceeding to adopt, even if we can be positively sure of a clear common duct.

Dr. Parkes, in conclusion, advises from his experience in this case the general practice of sounding the common duct through the external opening. He is quite sure that such procedure can be safely carried out, and equally certain that its adoption in his case resulted in relief that did not follow the removal of the calculi alone.

METEOROLOGICAL SUMMARY FOR JUNE AT THIS STATION.

| | |
|---|---|
| Highest Temperature, June 6th, . . . . . . . . . . . | 90.2 |
| Lowest Temperature, June 11th, . . . . . . . . . . . | 56.6 |
| Greatest Daily Range of Temperature, . . . . . . . . . | 19.5 |
| Least Daily Range of Temperature, . . . . . . . . . | 10.2 |
| Mean Daily Range of Temperature. . . . . . . . . . | 14.5 |
| Mean Daily Dew-point, . . . . . . . . . . . . . . | 65.4 |
| Mean Daily Relative Humidity, . . . . . . . . . . . | 70.9 |
| Prevailing Direction of Wind, . . . . . . . . . | Southeast. |
| Total Movement of Wind, . . . . . . . . . . . | 4.922 Miles. |
| Highest Velocity of Wind and Direction, . . . | 27 Miles, West. |
| Number of Foggy Days, . . . . . . . . . . . . . | None. |
| Number of Clear Days, . . . . . . . . . . . . . . | 11 |
| Number of Fair Days, . . . . . . . . . . . . . . | 14 |
| Number of Cloudy Days, . . . . . . . . . . . . . | 5 |
| Number of Days on which Rain or Snow fell, . . . . . | 9 |

THE INTERNATIONAL MEDICAL CONGRESS AND THE MEDICAL PROFESSION OF PHILADELPHIA.—A meeting of the members of the medical profession of Philadelphia, concerned in the organization of the International Medical Congress of 1887, was held at the Hall of the College of Physicians, on Monday, June 29th, Dr. Alfred Stillé in the chair. Dr. David W. Yandell, of Louisville, was present by invitation.

After hearing a report of the proceedings of the new committee, at the meeting held in Chicago last week, and after considering the changes in the organization which were made, including the restriction of the scope of the membership, by which a large proportion of the profession of the country would be excluded from the Congress, the following preambles and resolution were unanimously adopted:

WHEREAS, Certain serious changes have been recently effected in the preliminary organization and rules for the International Medical Congress of 1887, it has seemed desirable for the members of the General Committee and the officers of the Sections resident in Philadelphia to meet for consultation; and

WHEREAS, It has appeared that these changes are inconsistent with the original plan, and detrimental to the interests of the medical profession in America, and of the International Medical Congress; therefore, be it

*Resolved*, That we, the undersigned, consider that our duty to the profession and to ourselves requires us to decline to hold any office whatsoever in connection with the said Congress as now proposed to be organized:

| | |
|---|---|
| D. HAYES AGNEW, | S. WEIR MITCHELL, |
| ROBERTS BARTHOLOW, | WILLIAM F. NORRIS, |
| JOHN H. BRINTON, | WILLIAM OSLER, |
| CHARLES H. BURNETT, | JOHN H. PACKARD, |
| R. A. CLEEMANN, | THEOPHILUS PARVIN, |
| J. M. DACOSTA, | WILLIAM PEPPER, |
| LOUIS A. DUHRING, | EDWARD T. REICHART, |
| WILLIAM H. FORD, | ALBERT H. SMITH, |
| WILLIAM GOODELL, | ROBERT MEADE SMITH, |
| SAMUEL W. GROSS, | ALFRED STILLE, |
| ROBERT P. HARRIS, | GEORGE STRAWBRIDGE, |
| I. MINIS HAYS, | WILLIAM THOMSON, |
| WILLIAM W. KEEN, | JAMES TYSON, |
| JOSEPH LEIDY, | HORATIO C. WOOD, |

DAVID W. YANDELL.

THE INTERNATIONAL MEDICAL CONGRESS AND THE MEDICAL PROFESSION OF BALTIMORE.—In consequence of the dissatisfaction caused by the recent action of the new Committee on the Organization of the Ninth International Medical Congress, the subjoined paper has been signed by those whose names are appended:

WHEREAS, The new Committee on the Organization of the Ninth International Medical Congress, at its recent meeting, held in Chicago, made such changes in the arrangements for the Congress as, in our opinion, will mar its success, and will prove injurious to the interests of the medical profession, it is therefore

*Resolved,* That we, the undersigned, disapprove of the action of the Committee, and decline to accept the positions to which we have been appointed under it:

| | |
|---|---|
| I. E. ATKINSON, | RICHARD MCSHERRY, |
| S. C. CHEW, | F. T. MILES, |
| JULIAN J. CHISOLM, | ALLAN P. SMITH, |
| CHRISTOPHER JOHNSTON, | SAMUEL THEOBALD, |
| WILLIAM LEE, | L. MCLANE TIFFANY, |
| JOHN N. MACKENZIE, | H. P. C. WILSON. |

THE following letter from Dr. McHatton is published with the hope that the profession in the South will give him the desired aid:

MACON, GA., July, 1885.

DEAR DOCTOR:—I am anxious to make as complete an investigation of malarial Hematuria as possible. It is a subject that can only be studied to advantage by getting the opinions and clinical experience of practitioners throughout the State.

I hope to obtain your assistance in the following points: Clinical history, treatment and rate of mortality.

I will also be much obliged to you if you can send me specimens of urine from cases occurring in your practice during the coming summer and fall. Send two ounce bottles by mail or express at my expense. Have them as fresh as possible with the history of the case, and the period of the disease at which they were passed.

Yours truly,

H. MCHATTON, M. D.,

*Macon, Georgia.*

THE INTERNATIONAL MEDICAL CONGRESS AND THE MEDICAL PROFESSION OF BOSTON.—The following gentlemen have requested to have their names appended to the list of signers to the resolutions adopted in Boston, declining to hold office in the proposed Congress as now organized, which appeared in *The News* of last week, page 53 : O. W. Holmes, Wm. H. Baker, David W. Cheever, James C. White, William F. Whitney, Boston; G. P. Conn, Concord, N. H.; F. H. Gerrish, S. C. Gordon, Portland, Me.; E. P. Hurd, Newburyport, Mass.; Nathan Allen, Lowell, Mass.

THE Surgical Chair, manufactured by the Canton Surgical Chair Company, of Canton, Ohio, is the best chair of the kind we know. We would not be without it for double its cost.

THE INTERNATIONAL MEDICAL CONGRESS AND THE MEDICAL PROFESSION OF WASHINGTON.—At a meeting of medical gentlemen, held in Washington, D. C., July 11, 1885, the following preamble and resolutions were adopted:

WHEREAS, Certain changes have been made in the constitution and organization of the Ninth International Medical Congress which seem to us unwise, injurious, calculated to bring the profession into disrepute, and to endanger the success of the Congress : therefore

*Resolved*, That we, the undersigned, decline to hold any position under the said Congress as now organized.

| | |
|---|---|
| JOSEPH TABER JOHNSON, M.D. | S. C. BUSEY, M. D. |
| W. W. JOHNSTON, M.D. | H. C. YARROW, M.D. |
| SWAN M. BURNETT, M.D. | A. F. A. KING, M.D. |
| B. F. POPE, M.D., U. S. Army. | FRANK BAKER, M.D. |
| E. CARROLL MORGAN, M.D. | D. WEBSTER PRENTISS, M.D. |
| J. FORD THOMPSON, M.D. | S. O. RICHEY, M.D. |

D. L. HUNTINGTON, M.D., U. S. Army.

HOW TO AVOID NIGHT CALLS.—A story is going the rounds (who started it we do not know) at the expense of the young physician who is always so busy that he doesn't know what to do.

"I have got more business than I can attend to," boasted he to an old practitioner who knew he lied. "I had to get out of bed five times last night." "Why don't you buy some insect powder?" quietly asked the old doctor.—*Medical Age.*

THE TREATMENT OF EPISTAXIS.—Introduce into the nostril, to a considerable distance upward, a piece of fine sponge, cut to the size and shape necessary to enable it to enter without difficulty, previously soaked in lemon juice or vinegar and water. The patient is to be kept lying on the face for a length of time, with the sponge in place. This, says "Lyon médical," is the procedure employed by M. Siredey for controlling epistaxis in typhoid-fever patients.—*N. Y. Medical Journal.*

THE VALUE OF CASCARA SAGRADA IN CONSTIPATION.—At a recent meeting of the Staffordshire Branch of the British Medical Association, Dr. Reid read a paper upon the value of this drug, based on an analysis of thirty-three cases, of which he had taken careful notes. He found: 1, that the result was all that could be desired in twenty-seven cases of obstinate and habitual constipation, complicated in many cases by various forms of dyspepsia, and in many people usually of sedentary habits, more especially females; 2, that the effect was most beneficial in three cases of hemorrhoids; 3, that the drug was of no service, even in very large doses, in one case of obstinate constipation, although it did not cause pain; 4, that it had to be discontinued in two cases on account of its causing pain and sickness. The form of drug used was the fluid extract. The conclusions arrived at were as follows: 1. Cascara sagrada was a most useful remedy, both regarding its immediate effects and after-results in obstinate and chronic cases of constipation. 2. It was better to prescribe it in continuous small doses rather than in occasional large ones. 3. Cases were met with in which, even in large doses—at any rate, in the form of the fluid extract—the drug had not been of service. 4. No rule could be laid down by which one could ascertain previously whether the drug would suit or not; but, when pain was produced, in all probability it was owing to too large a dose being given. 5. It was of great service in cases of hemorrhoids when other aperients had failed.—*British Medical Journal.*

